# Chromosome-level assembly, annotation and phylome of *Pelobates cultripes*, the western spadefoot toad

**DOI:** 10.1093/dnares/dsac013

**Published:** 2022-05-18

**Authors:** Hans Christoph Liedtke, Fernando Cruz, Jèssica Gómez-Garrido, Diego Fuentes Palacios, Marina Marcet-Houben, Marta Gut, Tyler Alioto, Toni Gabaldón, Ivan Gomez-Mestre

**Affiliations:** Ecology, Evolution and Development Group, Department of Wetland Ecology, Estación Biológica de Doñana (CSIC), 41092 Sevilla, Spain; CNAG-CRG, Centre for Genomic Regulation (CRG), Barcelona Institute of Science and Technology (BIST), 08028 Barcelona, Spain; CNAG-CRG, Centre for Genomic Regulation (CRG), Barcelona Institute of Science and Technology (BIST), 08028 Barcelona, Spain; Barcelona Supercomputing Centre (BSC-CNS), 08034 Barcelona, Spain; Institute for Research in Biomedicine (IRB), The Barcelona Institute of Science and Technology (BIST), 08028 Barcelona, Spain; Barcelona Supercomputing Centre (BSC-CNS), 08034 Barcelona, Spain; Institute for Research in Biomedicine (IRB), The Barcelona Institute of Science and Technology (BIST), 08028 Barcelona, Spain; CNAG-CRG, Centre for Genomic Regulation (CRG), Barcelona Institute of Science and Technology (BIST), 08028 Barcelona, Spain; CNAG-CRG, Centre for Genomic Regulation (CRG), Barcelona Institute of Science and Technology (BIST), 08028 Barcelona, Spain; Universitat Pompeu Fabra (UPF), Barcelona, Spain; Barcelona Supercomputing Centre (BSC-CNS), 08034 Barcelona, Spain; Institute for Research in Biomedicine (IRB), The Barcelona Institute of Science and Technology (BIST), 08028 Barcelona, Spain; Catalan Institution for Research and Advanced Studies (ICREA), Barcelona, Spain; Ecology, Evolution and Development Group, Department of Wetland Ecology, Estación Biológica de Doñana (CSIC), 41092 Sevilla, Spain

**Keywords:** genome assembly, amphibia, phylome

## Abstract

Genomic resources for amphibians are still hugely under-represented in vertebrate genomic research, despite being a group of major interest for ecology, evolution and conservation. Amphibians constitute a highly threatened group of vertebrates, present a vast diversity in reproductive modes, are extremely diverse in morphology, occupy most ecoregions of the world, and present the widest range in genome sizes of any major group of vertebrates. We combined Illumina, Nanopore and Hi-C sequencing technologies to assemble a chromosome-level genome sequence for an anuran with a moderate genome size (assembly span 3.09 Gb); *Pelobates cultripes*, the western spadefoot toad. The genome has an N50 length of 330 Mb with 98.6% of the total sequence length assembled into 14 super scaffolds, and 87.7% complete BUSCO genes. We use published transcriptomic data to provide annotations, identifying 32,684 protein-coding genes. We also reconstruct the *P. cultripes* phylome and identify 2,527 gene expansions. We contribute the first draft of the genome of the western spadefoot toad, *P. cultripes*. This species represents a relatively basal lineage in the anuran tree with an interesting ecology and a high degree of developmental plasticity, and thus is an important resource for amphibian genomic research.

## 1. Introduction

Over the course of evolution, eukaryotic genomes have diverged enormously, resulting in a wide range of genome sizes across taxa.^1^^,^^2^ As genomes increase in size, they also change their structure, with the number of genes evolving much more slowly than changes in genomic architecture due to polyploidization, variation in transposable elements (TEs), intron frequency or abundance of repeat elements.^3^^,^^4^ Non-adaptive accumulation of TEs or neutral processes associated with population dynamics may have played a role in shaping genome size and complexity in at least some eukaryotes.^3^^,^^5^ Nevertheless, variation in genome size and structure seems to also be associated with life-history traits, reproductive mode or developmental rate.^6–10^ However, these relationships are only poorly understood, in part due to limited genomic resources on a wide spectrum of taxa.

Amphibians constitute an ideal study group to expand our genomic knowledge because they present vast diversity in reproductive modes,^11^^,^^12^ including losses and gains of entire life stages, occupy most ecoregions of the world, are morphologically highly diverse, and constitute the most threatened group of vertebrates worldwide.^13^^,^^14^ They also have immense potential in pharmaceutical sciences and biotechnology.^15–17^ Unfortunately, amphibian genomes tend to be large, with some salamander species possessing among the largest genomes known of any vertebrate.^18^ Perhaps because of this, they have received less attention in comparison to other taxa such as mammals, birds or non-avian reptiles. At the time of writing, only 29 species of amphibians (∼0.3% of described species) with reference genomes were listed on the National Center for Biotechnology Information (NCBI; https://www.ncbi.nlm.nih.gov/, 1 December 2021, date last accessed) database, compared to 529 bird species (∼4.7%), 461 mammal species (∼7.1%) and 70 non-avian reptiles (∼0.6%). Fortunately, improved massive parallel sequencing techniques and the combination of different sequencing approaches—including long-read technologies—with state-of-the-art assembly algorithms are facilitating the sequencing and assembly of complex amphibian genomes.^19^^,^^20^ Last year alone (up to 1 December 2021), 15 new amphibian reference genomes have been listed on the NCBI database, 10 at the chromosome level.

**Figure 1:**
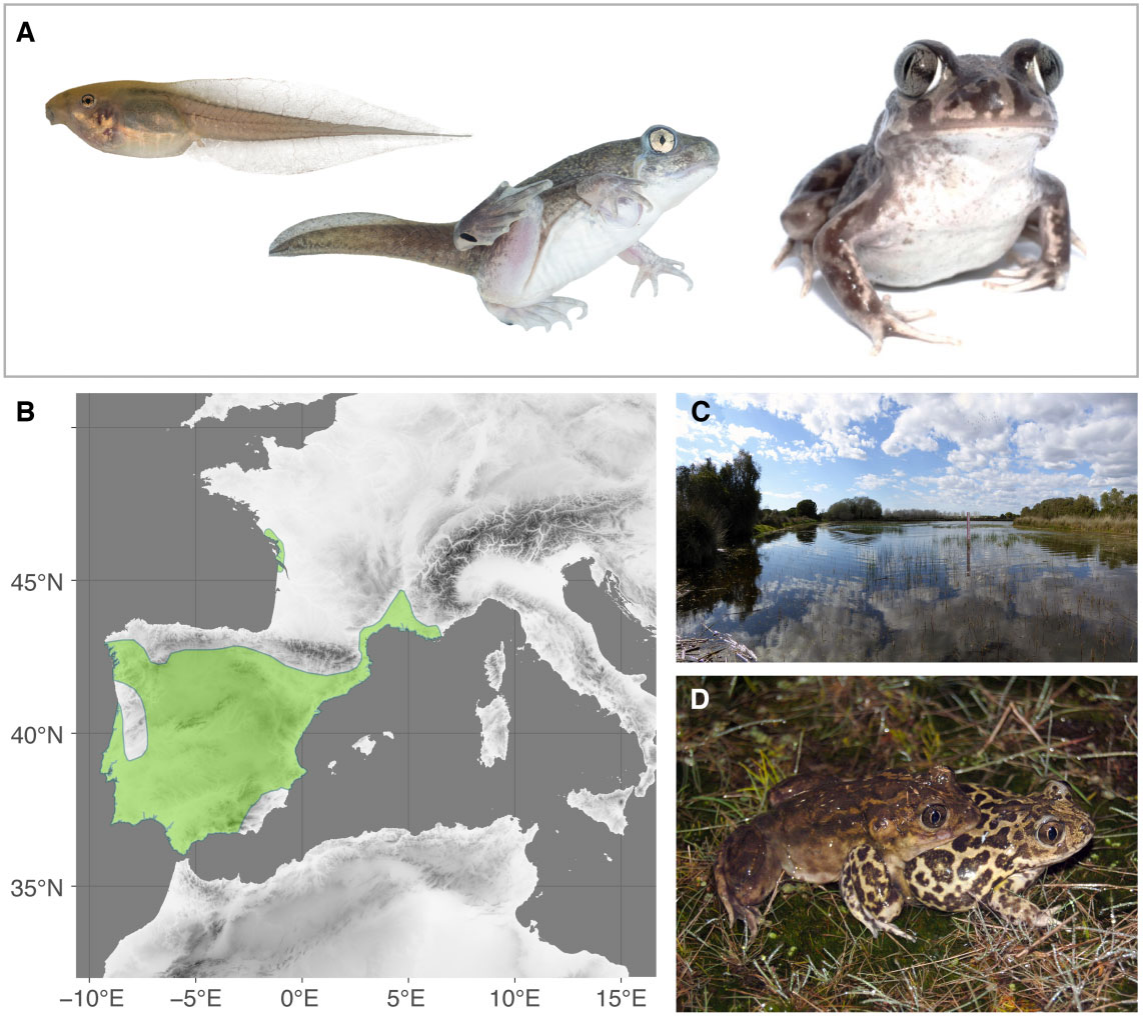
Biology of the western spadefoot toad, *P. cultripes*: (A) Complex life cycle with a larval, metamorphic and adult phase. (B) Current distribution range for the species, from the IUCN Red List (https://www.iucnredlist.org/, 11 January 2022, date last accessed). (C) Example of breeding habitat for the species; a temporary pond at Doñana National Park, Huelva, Spain. (D) Amplectant pair of *P. cultripes* from Doñana National Park.

Here, we contribute the first draft of the genome of the western spadefoot toad, *Pelobates cultripes* (Cuvier, 1829; Fig. 1). This species forms a basal lineage within the Pelobatidae family,^21^ which in turn is a relatively deep diverging clade in the anuran tree.^22^ It inhabits south-western Europe, including most of the Iberian Peninsula reaching parts of southwestern France.^23^^,^^24^ As adults, *P. cultripes* are medium sized anurans (40–120 mm, snout-vent length), with sexual size dimorphism in favour of females. This species breeds once a year, laying large clutches (1,300–4,000 eggs) in temporary ponds where the larvae develop for an extended period of time until they reach metamorphosis, usually at a large size.^25^^,^^26^ The large larvae of this species have a strong effect on the aquatic communities they inhabit, where they can reach high densities and cover long distances,^27^ causing a big herbivorous impact on aquatic macrophytes^28^^,^^29^ and zooplankton.^28^*Pelobates cultripes* larvae are developmentally plastic, being capable of accelerating their development by ∼30% when they perceive risk of desiccation from pond drying, at the expense of metamorphosing at a smaller size and increased oxidative stress.^30^^,^^31^ These larvae achieve remarkable developmental acceleration elevating their level of corticosterone and both thyroid hormone and thyroid hormone receptor, as well as increasing their metabolic rate and lipid catabolism.^31–33^

*Pelobates cultripes* is known to have a diploid genome with 13 chromosome pairs (2*n* = 26). All chromosomes have two arms and comprise 6 large and 7 small pairs.^34^^,^^35^ C-bands are present in several chromosomes, ranging from centromeric to pericentric and telomeric, with several chromosomes markedly submetacentric and one pair being almost completely heterochromatic. There is no evidence for sexually dimorphic chromosomes in this species. Instances of both heterogametic males and females are common in amphibians, although female heterogamety is thought to be ancestral.^36^^,^^37^

## 2. Materials and methods

### 2.1. Biological material

We collected a mature *P.cultripes* female near a pond in Valdemanco, central Spain (Laguna de Valdemanco, Madrid, Spain), and transported it to the Doñana Biological Station (Seville) where it was euthanized to harvest different organs and tissues. The procedure was approved under the Institutional Animal Care and Use Committee (IACUC) permit no. 18_09 CEBA_EBD. Upon dissection, tissues were snap frozen in liquid nitrogen and stored at −80°C until DNA extraction. DNA for whole-genome sequencing was extracted from muscle tissue using standard phenol-chloroform procedure. Extracted nucleic acid quality and quantity were assayed on a Bioanalyzer 2100 (Agilent Technologies, Santa Clara, CA, USA). This specimen, from which the present draft genome was generated, has been assigned the Tree of Life identifier *aPelCul1* (https://id.tol.sanger.ac.uk/).

### 2.2. Library construction and sequencing

Our sequencing strategy was to combine complementary sequencing technologies to achieve sufficient coverage and obtain robust scaffolding to generate a good assembly. We began by generating short paired-end reads (2 × 250 bp) and long-insert mate pair (MP) reads (insert sizes of 3,000 and 8,000 bp). Illumina sequences were then complemented with Oxford Nanopore sequencing [Oxford Nanopore Technologies (ONT), Oxford, UK] to generate a draft assembly. We then used Chicago + Dovetail Hi-C libraries in combination with Hi-Rise scaffolding to improve the contiguity and accuracy of the assembly (Dovetail Genomics, Scotts Valley, CA, USA). Below we detail each of these steps.

The short-insert paired-end libraries for whole-genome sequencing were prepared with KAPA HTP/LTP Library Preparation kit (KAPA Biosystems, Cape Town South Africa) with some modifications. In brief, 4.0 μg of genomic DNA was sheared on a Covaris™ E210 (Covaris Inc., Woburn, MA, USA), the fragmented DNA was size-selected on 1% agarose gels to obtain three duplicated PE libraries with incremental insert sizes. The fragments were end-repaired, adenylated and ligated to NEXTflex-96™ DNA Barcodes (Bioo Scientific Corporation, Austin, TX, USA) paired-end adaptors. The libraries were quality controlled on an Agilent 2100 Bioanalyzer with the DNA 7500 assay for size and the concentration was estimated using quantitative PCR with the KAPA Library Quantification Kit Illumina^®^ Platforms. The three paired-end library sizes were 480, 600 and 700 bp. In order to obtain the required input for DISCOVAR *de novo* (https://software.broadinstitute.org/software/discovar/blog/), the 480 bp library was selected for sequencing on Hiseq 2500 (Illumina) in rapid mode with 2 × 251 bp paired-end read length, according to standard Illumina operation procedures. The 480 bp insert library was chosen because higher insert sizes tend to result in biased sequencing and because the DISCOVAR *de novo* algorithm is optimized for 250 bp read-length paired-end libraries with some degree of overlap. A total of 397 Gb of raw sequence were produced. Primary data analysis was carried out with the standard Illumina pipeline (RTA 1.18.66.3). MP libraries (3 and 8 kb fragment sizes) were constructed according to the Nextera Mate Pair Preparation protocol (Illumina Inc.), and sequenced on the HiSeq 2500 platform in 2 × 150 bp read length runs.

Pre-processing sequence reads before assembly involved detection and trimming of Illumina adapter sequences, as well as quality scoring and trimming using the *cutadapt* tool in the Trim Galore! wrapper script^38^ (http://www.bioinformatics.babraham.ac.uk/projects/trim_galore/). The linker sequence present in the MP sequences was also removed with *cutadapt*. Overlapping reads derived from shorter fragments were merged using FLASH.^39^ Then, all reads were filtered by mapping, using gem-mapper,^40^ with up to 2% mismatches, against a contamination database that included phiX, Univec sequences, *Escherichiacoli*, the complete mitochondrial genome of *P. cultripes* (NC_008144.1) and various contaminants detected in more than 0.01% of the reads using Kraken (v0.10.5-beta).^41^ Before hybrid *de novo* assembly (see below), contaminants were filtered out from reads from both Paired-End (PE480) and MP (MP3000 and MP8000) libraries, but without removing their adaptors. In order to optimize the *de Bruijn* graph construction,^42^ the clean Illumina PE reads were also trimmed to 150 bp using FASTX toolkit v.0.0.13 (http://hannonlab.cshl.edu/fastx_toolkit/) regardless of their mean quality, because we have found that the last 50–100 cycles consistently produce lower base qualities. This hard trimming reduced the sequencing coverage, from 140× to 80×, but increased the mean base quality of the PE reads and therefore improved the superReads construction with MaSuRCA (see Section 2.3).

Nanopore sequencing was performed on an Oxford Nanopore Technologies (ONT) MinION Mk1B device. Because of the rapid progression of the technology during the project we used four different chemistry versions for library preparation [SQK-LSK 308 (1D2), SQK-LSK 108 (1D) and SQK-LSK 109 (1D) and SQK—RAD 004 (Rapid chemistry)] (ONT) and also different flow cell versions [FLO-MIN106 (R9.4 or R9.4.1) and FLO-MIN107 (R9.5)] (ONT). To prepare 1D, 1D2 and Rapid chemistry genomic libraries we used the same extracted genomic DNA from *P. cultripes* used for Illumina sequencing. For the ligation type of sample preparation, we employed sequencing kits SQK-LSK308, SQK-LSK108 and SQK-LSK109. For these library types, the starting material was 4–6 μg of genomic DNA processed without any fragmentation. Subsequently, the genomic DNA was repaired using NEBNext FFPE DNA Repair Mix (New England Biolabs, NEB) and purified with 0.4× AMPure XP Beads (Beckman Coulter Life Sciences). The second repair combined end-repair and dA-tailing [NEBNext UltraII End Repair/dA-Tailing Module (NEB)], followed by purification with 1× AMPure XP Beads. For libraries prepared with the SQK-LSK108 and 109 kit, the adapter mix 1D (ONT) was ligated to the purified DNA using the NEB Blunt/TA ligase Master Mix. For libraries prepared with the SQK-LSK308 kit, the adapter mix II for sequencing (ONT) was ligated to the purified DNA and, after a 0.4× AMPure XP Beads purification, the 1D2 Adapter (ONT) was also ligated. These two consecutive ligation steps were performed using the NEB Blunt/TA ligase Master Mix. For all ‘ligation type’ libraries, the ligation of the adapters was followed by a final purification with 0.4× AMPure XP Beads, then washed with ABB buffer (ONT) and eluted in Elution Buffer (ONT).

The sample preparation for the Rapid library type followed the protocol in the Rapid Sequencing kit SQK-RAD004. Briefly, ∼400 ng of purified DNA was tagmented with the Fragmentation Mix (ONT) and the Rapid Adapters (ONT) were added along with Blunt/TA Ligase Master Mix. The sequencing run of the SQK-LSK308 library was performed on a FLO-MIN107 flow cell while SQK-LSK108; SQK-LSK109 and SQK–RAD004 libraries were sequenced on FLO-MIN106 flow cells. The different flow cells were connected to a MinION Mk1B instrument for sequencing. In brief, first the MinKNOW interface QC (ONT) was run in order to assess the flow cell quality and this was followed by the flow cell priming. Once the libraries were loaded into the flow cells, the sequencing data were collected for 48 h. The quality parameters of the sequencing runs were monitored using the MinKNOW platform.

Nanopore data were base-called with Albacore v2.3.3 (ONT). Reads with the following criteria were filtered out: average reported base quality per read *Q* < 7, length <1 Kb or matching our ONT contamination database. This database was built by adding the control Sequence (lambda phage 3.5 Kb) to the database used for pre-processing the Illumina reads.

A Chicago library was prepared following standard procedure.^43^ Briefly, 500 ng of high molecular weight genomic DNA from the same individual was extracted and reconstituted into chromatin *in vitro* and fixed with formaldehyde. Fixed chromatin was digested with DpnII, and the 5′ overhangs were filled in with biotinylated nucleotides, and the free blunt ends were ligated together. After ligation, crosslinks were reversed and the DNA purified from protein. Purified DNA was treated to remove biotin that was not internal to ligated fragments. The DNA was then sheared to ∼350 bp mean fragment size and sequencing libraries were generated using NEBNext Ultra enzymes and Illumina-compatible adapters. Biotin-containing fragments were isolated using streptavidin beads before PCR enrichment of each library. The Chicago library was sequenced on an Illumina HiSeq X to produce 194 million 2 × 150 bp paired-end reads.

To improve the contiguity of the draft *P. cultripes* genome, we also incorporated Hi-C sequencing. Three Hi-C libraries were prepared due to the size and expected complexity of the *P. cultripes* genome, as seen in previously sequenced amphibian genomes. Hi-C libraries were prepared in a similar manner as described in Lieberman-Aiden *et al*.^44^ Briefly, for each library, chromatin was fixed in place with formaldehyde in the nucleus and then the fixed chromatin was digested with DpnII, the 5′ overhangs filled in with biotinylated nucleotides, and then free blunt ends were ligated. After ligation, crosslinks were reversed and the DNA purified from protein. Purified DNA was treated to remove biotin that was not internal to ligated fragments. The DNA was then sheared to ∼350 bp mean fragment size and sequencing libraries were generated using NEBNext Ultra enzymes and Illumina-compatible adapters. Biotin-containing fragments were isolated using streptavidin beads before PCR enrichment of each library. The libraries were sequenced on an Illumina HiSeq X to produce 259 million 2 × 150 bp paired-end reads.

### 2.3. Genome assembly

A flowchart of the assembly approach is provided as Supplementary Fig. S1 and can be summarized in six steps. (i) A preliminary draft genome assembly was obtained using DISCOVAR de novo v52488^45^ to assemble pre-processed PE480 2 × 250 bp reads and subsequent scaffolding of non-circular contigs with BESST v2.2.5,^46^ using the Illumina pre-processed PE480, MP3000 and MP8000 libraries. The contiguity of this draft assembly (scaffold N50 of 48.16 kb) was high enough for re-mapping and estimating the mean and standard deviation of fragment size for each Illumina sequencing library. Thus, the fragment size distributions were estimated by re-mapping with GEM-mapper build 1.375,^40^ allowing calculation of mean fragment size and deviation for each input library usedby MaSuRCA (see below). (ii) We obtained a first hybrid genome assembly with MaSuRCA v3.2.8^47^^,^^48^ using hard-trimmed PE 2 × 150 bp (80×), clean MP (41×) and pre-processed Nanopore reads (21.66×) to construct mega-reads and assemble them with CABOG v6.2.^49^ MaSuRCA estimated the genome size to be 2,825,570,248 by using *jellyfish*^50^ to extract 31-mers from all Illumina reads. (iii) We obtained, in parallel, a second hybrid assembly by again assembling the mega-reads (17.92× and N50 = 5.84 kb) with Flye v2.3.7^51^ using a minimum overlap of 2 kb (–m = 2000), genome size 2.82 Gb (-g 2825570248) and no polishing iterations (-i = 0). (iv) Both hybrid assemblies were merged using the *merge_scaffolds.sh* script included in MaSuRCA v3.3.2b with parameters -r masurca.scaffolds.fa -q flye.scaffolds.fa -t 48 -i 99 -o 1,000 -g 5,000 -G 10,000, where -i stands for identity, -o overhang, -g minimum gap and -G maximum gap. This procedure closed 1,123 gaps in the MaSuRCA assembly with the scaffolds produced with Flye. The merged hybrid assembly had contig N50 = 156.96 kb and scaffold N50 = 249.48 kb (Supplementary Table S1). (v) The merged assembly was used as input for Chicago/Hi-Rise^43^ and Dovetail/Hi-C by Dovetail Genomics, LLC (Scotts Valley, CA, USA). The Chicago^®^*invitro* proximity ligation library was sequenced, generating 194 million read pairs (2 × 150 bp). The input *de novo* assembly, Chicago library reads, and Dovetail HiC library reads were used as input data for *HiRise*, a software pipeline designed specifically for using proximity ligation data to scaffold genome assemblies.^43^ An iterative analysis was conducted. First, Chicago library sequences were aligned to the draft input assembly using a modified SNAP read mapper (http://snap.cs.berkeley.edu). The separations of Chicago read pairs mapped within draft scaffolds were analysed by HiRise to produce a likelihood model for genomic distance between read pairs, and the model was used to identify and break putative misjoins, to score prospective joins, and make joins above a threshold, producing 30,604 breaks and 13,294 joins. After aligning and scaffolding Chicago data, Dovetail HiC library sequences were aligned and scaffolded following the same method, allowing the introduction of 3,561 additional breaks and 36,279 new joins during the Hi-Rise™ scaffolding. The resulting Dovetail assembly (internal project name: *Pcu21*) was highly contiguous, with N50 330.12 Mb and 14 Superscaffolds (Supplementary Table S1). (vi) We collapsed the assembly with Purge Haplotigs v1.1.0^52^ in order to avoid the inclusion of redundant scaffolds corresponding to alternative haplotigs. The fully pre-processed PE480 reads were mapped against *Pcu21* with BWA MEM v.0.7.7^53^ and the -M option to discard mappings of chimeric reads (internal project name: *Pcu22*; Supplementary Table S1). Based on these mappings, we first plotted the coverage distribution with *purge_haplotigs hist* identifying three depth cut-offs: low = 13, midpoint = 85 and high = 234. Then scaffolds were flagged as ‘junk’ or ‘suspect’ based on their coverage and the cut-offs used. The assembly was collapsed, discarding a total of 12,224 redundant scaffolds, and had a total length of 3,094,940,563. Finally it was named and publicly released as *aPelCul1.1*.

To evaluate the final *aPelCul1.1* assembly we ran BUSCOv5.2.2 and scanned the genome assembly to detect contaminants using BlobToolsv1.1.^54^ BUSCO was run in genome and transcriptome mode against tetrapoda_odb10 (5,310 BUSCOs). BlobTools was run with the recommended *blastn* parameters (e-value 10e-25 -max_target_seqs 25 -culling_limit 2) against NCBI nucleotide collection (*nt* database updated on 30 September 2021) and found no evidence for contamination, likely due to the thorough decontamination of the input reads.

### 2.4. Genome annotation

A flowchart of the annotation process is shown in Supplementary Fig. S2. Repeats and low complexity DNA sequences in the *P. cultripes* genome assembly were annotated with RepeatMasker v4-0-7^55^ using the Amphibia repeat library present in Repbase v20170127. Next, we made a new repeat library specifically for our assembly with RepeatModeler v1.0.11 (http://www.repeatmodeler.org). After excluding those repeats that were part of repetitive protein families (performing a BLAST search against Swissprot) from the resulting library, we ran RepeatMasker again with this new library in order to annotate the specific repeats. We then annotated genes in the assembly by combining transcript alignments, protein alignments and *ab initio* gene predictions as follows.

First, RNAseq data from a *de novo* transcriptome assembly for *P. cultripes* (SRA: SRP161446^56^) were aligned to the genome with STAR v-2.7.2a.^57^ Transcript models were subsequently generated using Stringtie v1.0.4^58^ and consensus assemblies were produced with PASA (Programme to Assemble Spliced Alignments) v2.3.3.^59^ We then ran *TransDecoder*^60^ on the PASA assemblies to detect coding regions in the transcripts. Second, the complete *Xenopus laevis* and *Xenopus tropicalis* proteomes were downloaded from Uniprot in March 2020 and aligned to the genome using Spaln v2.2.2.^61^*Ab initio* gene predictions were performed on the repeat-masked assembly with three different programmes: GeneID v1.4,^62^ Augustus v3.2.3^63^ and Genemark-ES v2.3e^64^ with and without incorporating evidence from the RNAseq data. The gene predictors were run with the available human parameters, except Genemark, which runs in a self-trained manner. Finally, all the data were combined into consensus CDS models using EvidenceModeler-1.1.1.^59^ Additionally, UTRs and alternative splicing forms were annotated through two rounds of PASA annotation updates. Functional annotation was performed on the annotated proteins with Blast2GO v1.3.3.^65^ First, a Protein BLAST^66^ search was made against the nr database (last accessed March 2020). Furthermore, InterProScan v5.21.60^67^ was run to detect protein domains on the annotated proteins. All these data were combined by Blast2GO which produced the final functional annotation results.

The annotation of ncRNAs was produced as follows. First, the programme cmsearch v1.1,^68^ which comes with Infernal,^69^ was run against the RFAM^70^ database of RNA families v12.0. Also, tRNAscan-SE v1.23^71^ was run in order to detect transfer RNA genes present in the genome assembly. To detect lncRNAs, we selected those PASA assemblies that had not been included in the annotation of protein-coding genes in order to get all those expressed genes that were not translated into proteins. Finally, those PASA assemblies without protein-coding gene annotation that were longer than 200 bp and whose length was not covered in at least 80% by a small ncRNA were incorporated into the ncRNA annotation as lncRNAs. The resulting transcripts were clustered into genes using shared splice sites or significant sequence overlap as criteria for designation as the same gene.

### 2.5. Phylome reconstruction

We reconstructed the phylome of the western spadefoot toad *P. cultripes* to contextualize its genome evolution in a comparative framework with other available genomes. In addition to this species, we selected representative amphibian species based on phylogenetic position, genome completeness and quality, as well as availability of genome annotation. The species included (Supplementary Table S2) were: *Rhinatrema bivittatum* (Gymnophiona, Rhinatrematidae), *Geotrypetes seraphini* (Gymnophiona, Dermophiidae), *X.tropicalis* (Anura, Pipidae), *Nanorana parkeri* (Anura, Dicroglossidae), *Lithobates catesbeianus* (Anura, Ranidae), *Spea multiplicata* (Anura, Scaphiopodidae), *Leptobrachium ailaonicum* (Anura, Megophryidae) and *Leptobrachium leishanense* (Anura, Megophryidae). As outgroups, we selected four taxa spanning several classes across the Chordata phylum: *Lepisosteus oculatus* (Actinopterygii, Lepisosteidae), *Latimeria chalumnae* (Coelacanthiformes, Latimeriidae), *Gallus Gallus* (Aves, Phasianidae) and *Homo Sapiens* (Mammalia, Hominidae).

For each protein annotated in the *P. cultripes* genome assembly (see Section 2.4) a BLASTP v2.5.0+ search was performed against the proteome database generated from all the species selected for the phylome reconstruction (a total of 287,300 proteins) to retrieve a set of proteins with a significant similarity (e-value < 1e−05). Only sequences that aligned with a continuous region longer than 50% of the query sequence were selected. The top 150 hits per query protein were kept.

Sets of homologous protein sequences were aligned using three different programmes: MUSCLE v3.8.1551,^72^ MAFFT v7.407^73^ and KALIGN v2.04.^74^ Alignments were performed in forward and reverse direction and the six resulting alignments were combined using M-COFFEE from the T-COFFEE package v12.0.^75^ The resulting alignment was trimmed using trimAl v1.4.rev15^76^ using a consistency cut-off of 0.1667 and a gap score cut-off of 0.1.

IQ-Tree v1.6.9^77^ was used to reconstruct the phylogenetic tree. Model selection was performed as implemented in iqtree and the models were limited to DCmut, JTTDCMut, LG, WAG and VT. Categories for the FreeRate model were set to range between 4 and 10. The best model according to the Bayesian Information Criterion (BIC) was selected. 1,000 rapid bootstraps were calculated. The reconstructed phylome, including all gene trees and alignments, as well as orthology and paralogy relationships can be interactively browsed or downloaded from PhylomeDB^78^ with the PhyID code 44.

To predict the relationships of orthology and paralogy across genes and species, we used the ETE3 package.^79^ This method takes into account evolutionary events such as speciation or gene duplication. In order to do so, it considers a node susceptible to be a speciation node if there are no overlapping species at either side of the node. Otherwise, it is considered a duplication node. Orthologues were inferred from speciation events assuming that genes derived from a speciation event are orthologous.^80^

We identified genes that had undergone a duplication event in different lineages, and estimated their relative age based on information of species that diverged prior and after the duplication node.^81^ FatiGO^82^ was used for GO enrichment analysis by comparing the annotation of proteins involved in a duplication event at a given age vs. all others for the seed genome. In order to account for the effect of TEs in amphibian genomes, we ran HMMER v3.3.2^83^ to detect proteins encoded by TEs. Those proteins were filtered out of the analysis, and then proceeded to recalculate all metrics and statistics mentioned above. Tree maps of significantly enriched terms were generated with REVIGO.^84^

All orphan genes, i.e. genes that did not have any BLAST hit during the all-vs-all comparison were interpreted as *P. cultripes* specific genes. These genes were then scrutinized via functional annotations with InterProScan v.5.47.^67^

## 3. Results and discussion

### 3.1. Genome assembly and annotation

**Figure 2:**
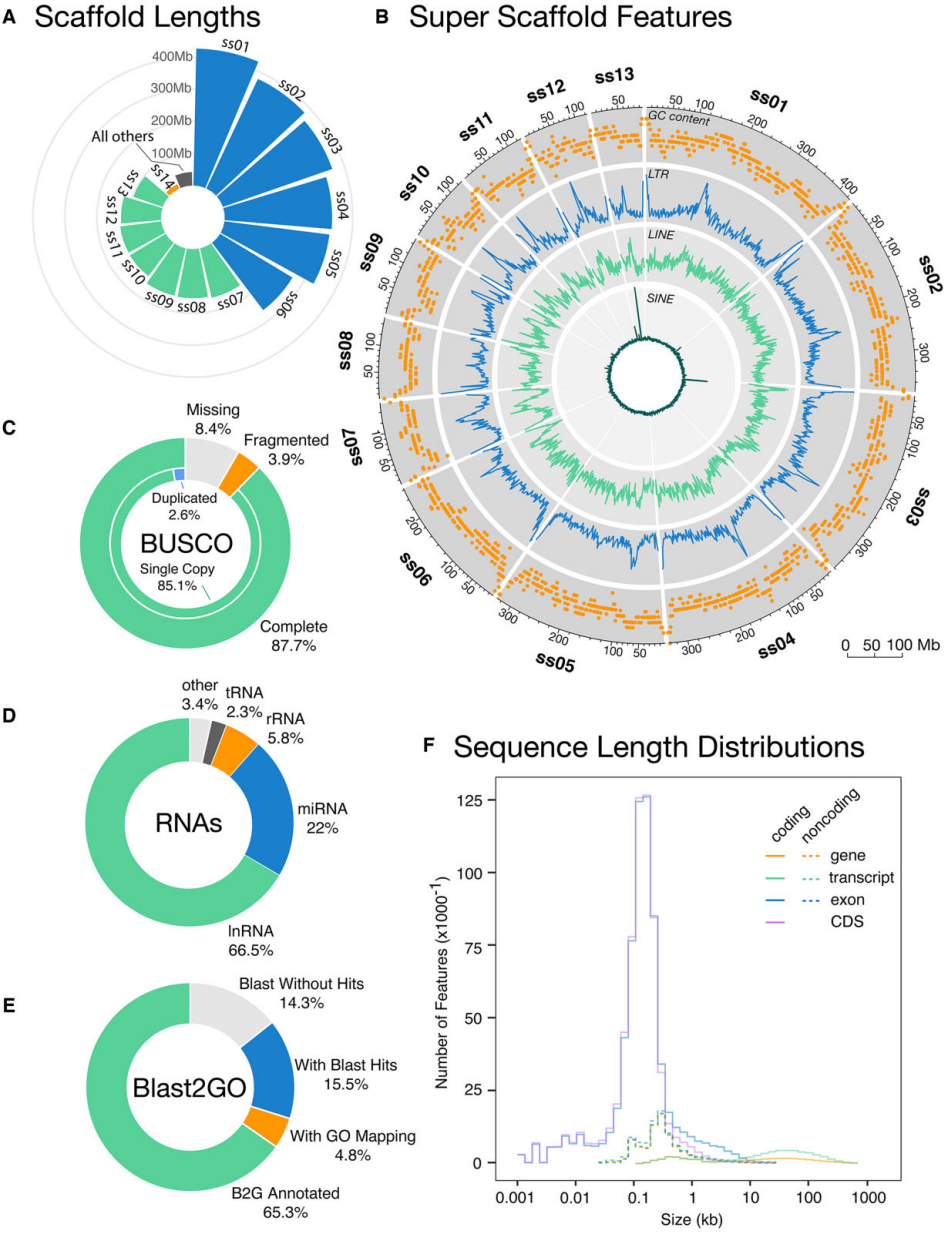
Genome assembly and annotation statistics. (A) Size (in Mb) of assembly superscaffolds 1–14, compared with the combined size of all remaining 3,560 scaffolds. Superscaffolds (ss) 1–6 (blue) are large, superscaffolds 7–13 (green) are smaller and superscaffold 14 (orange) is the smallest superscaffold. (B) Circular visualization of genomic features of superscaffolds 1–13. (A and B) show GC content (%) and number of LTR, LINE and SINE elements per ∼2 Mb window along each superscaffold. (C) Pie chart showing the percentages of complete BUSCO genes (distinguishing between single-copy and duplicates) compared with fragmented and missing genes. (D) Pie chart showing the proportions of RNAs annotated as lncRNA, miRNA, rRNA and tRNA compared with all other types combined. (E) Pie chart showing the percentage of sequences with functional annotations (from Blast2GO) in comparison to those not annotated due to missing results in the blast, mapping or annotation steps. (F) Size (in kb) distribution of coding elements (genes, transcripts, exons and CDS) and non-coding elements (genes, transcripts and exons). (A color version of this figure appears in the online version of this article.)

Our final genome assembly is highly contiguous, achieving a contig N50 of 130 Kb and scaffold N50 330 Mb (Table 1 and Supplementary Table S1). The assembly spans 3.09 Gb and we assembled 14 superscaffolds which make up 98.7% of the total assembly (Fig. 2A andSupplementary Table S3). The 13 superscaffolds (427.9–96.7 Mb) likely correspond to the 13 chromosomes observed in the *P. cultripes* karyotype. The small 14th superscaffold (14.4 Mb) may be a microchromosome, composed largely of telomeric region, as the contact map shows few contacts of this scaffold with the superscaffolds, and those contacts are restricted to their telomeric regions (Supplementary Fig. S4). Therefore, in congruence with the published karyotypes (e.g. reference^34^), we recover six large chromosomes (>250 Mb) and seven smaller chromosome scaffolds (96.7–158.1 Mb; Fig. 2A). The assembly contains 87.7% complete BUSCO genes (85.1% in Single-Copy and 2.6% Duplicated; Fig. 2C) and 8.4% missing from the total 5,310 in tetrapoda_odb10. Consistently with this, the results obtained in transcriptome mode using all the annotated transcripts (see below) also found 87.9% complete genes. We consider this to be an adequate level of gene completeness, *au pair* with that of the recent *Rana temporaria* chromosome-level assembly (90.7% completeness)^85^ and that of *S.multiplicata* (89.8% completeness).^86^

**Table 1:** *Pelobates cultripes* genome assembly scaffold statistics

	Size (bp)
N50	330,123,935 (*n* = 5)
N60	263,823,987 (*n* = 6)
N70	158,080,380 (*n* = 7)
N80	151,236,284 (*n* = 9)
N90	128,850,574 (*n* = 11)
N100	200 (*n* = 3574)
Max scaffold length	427,870,202
Mean scaffold length	865,959.87
Mean SuperScaffold length	218,079,234

Sample sizes (*n*) indicate number of scaffolds superseding the corresponding size.

In total, we annotated 32,684 protein-coding genes, producing 59,231 transcripts (1.81 transcripts per gene) and encoding 51,671 unique protein products. The annotated transcripts contain 11.22 exons on average, with 81.0% of them being multi-exonic (Table 2). Coding genes have a mean length of 39,593.05 bases and exons have a mean length of 277.76 bases (Fig. 2F). In addition, 80,638 non-coding transcripts were annotated, of which 53,652 (66.5%) and 26,986 (33.5%) are long- and short-non-coding RNA genes, respectively (Fig. 2D). Of the latter, 65.7% are microRNAs.

**Table 2:** *Pelobates cultripes* genome annotation statistics

	aPelCul1.2 Annotation
Number of protein-coding genes	32,684
Median gene length (bp)	8,947
Number of transcripts	59,231
Number of proteins	51,671
Coding GC content (%)	45.8
Median UTR length (bp)	1,571
Median intron length (bp)	1,139
Exons per transcript	11.22
Transcripts per gene	1.81

The mean GC content of the genome is 40.7%, whereas in the coding regions it reaches 45.8% (Table 2). Consistently with the distribution of isochores invertebrates, GC content also increases at the ends of each super scaffold with a further spike in central regions (Fig. 2B). These spikes are often associated with telomeres and in *P. cultripes* (aPelCul1.1.) also coincide with an increased concentration of long terminal repeats (LTRs) and to a lesser extent long interspersed nuclear elements (LINEs). Therefore, concentrations of these elements are likely to correspond to the C-bands observed in the karyotype. While in general the presence of SINE elements (short interspersed nuclear elements) along the genome is very low (0.04%), we did detect several regions with a higher abundance of SINE elements, mainly in Chromosomes 3 and 13 (Fig. 2B). Interestingly, these peaks are made up of tandemly repeated SINE elements, with different sequences expanded on each of the chromosomes. After extracting the sequence of these SINE elements, we tried to find them with RepeatMasker in the genomes of *Leptobrachium leishanensis* and *Leptobrachium boringii*, but failed to locate any expansion of these elements. Hence, we conclude that these might be more recent expansions.

We provide functional labels [Gene Ontology (GO) terms] for 65.3% of all annotated proteins (Fig. 2E). A further 4.8% of the proteins contained known protein domains (Interproscan) and an additional 15.5% gave significant BLAST hits but were not incorporated into the final annotation because they did not pass the BLAST2GO quality thresholds. 14.3% of the proteins did not have any functional hit.

### 3.2. Effect of TEs and gene expansions

A total of 29,289 gene trees were reconstructed for the western spadefoot toad phylome. Of these, 8,371 trees (28.6% of the total) were flagged as containing putative TE-related proteins, based on the presence of protein domains (see Section 2). In addition, we computed duplication rates—average number of duplication events per gene in a given lineage—taking into account all genes after filtering proteins encoded by TE and whether expansions were considered or not. Duplication rates were fairly low in both conditions, reporting the highest frequency at the western spadefoot toad terminal lineage (i.e. species-specific). Out of the remaining trees after TE filtering, 148,976 orthologues to other species present in the phylome were inferred.

**Figure 3:**
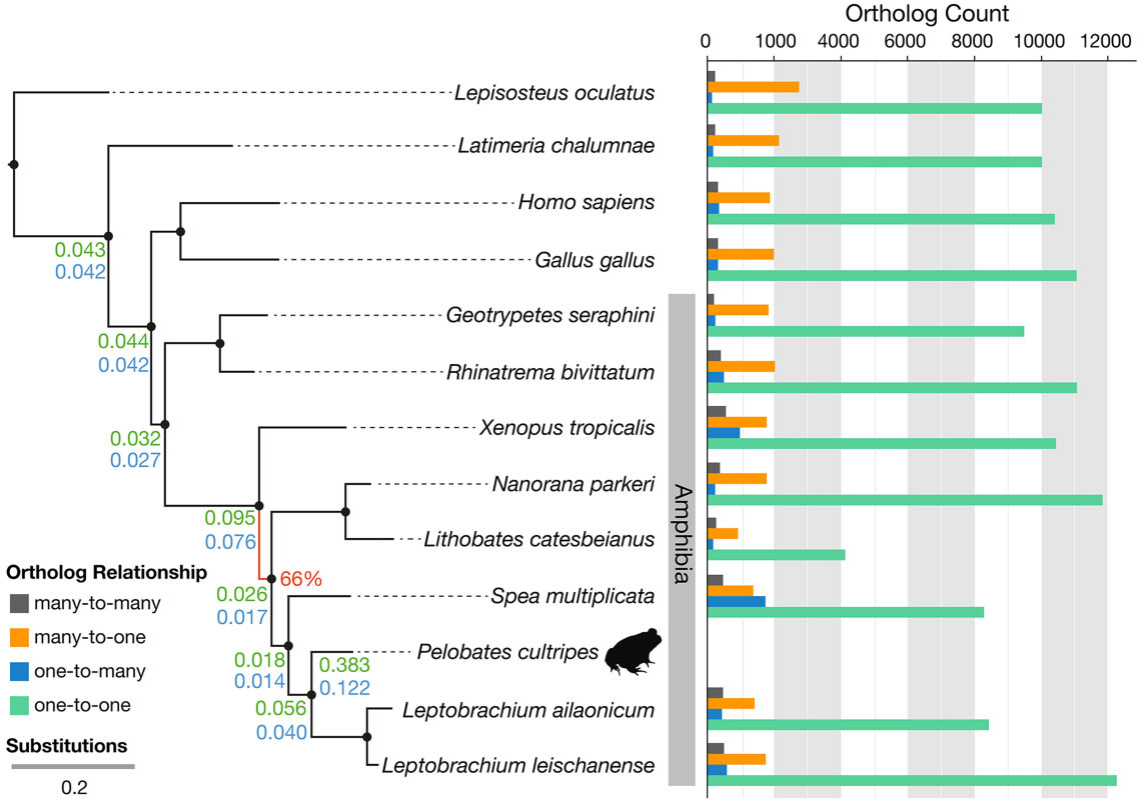
Species tree reconstructed from a concatenated alignment of 156 single-copy proteins present in the 13 species included in the phylome reconstruction. Branch lengths represent substitution rate. All branches had bootstrap values of 100% except one, indicated in red (66%). Branches leading to the focal species (*P. cultripes*) are annotated with duplication rates obtained when including (green, upper values) or excluding (blue, lower values) gene expansions, after filtering out TEs. The bar chart shows orthologue counts for each species in relation to the focal species, distinguishing between four possible relationships (many-to-many, many-to-one, one-to-many, one-to-one). (A color version of this figure appears in the online version of this article.)

The majority of the orthology relationships detected were one-to-one (Fig. 3). We then focussed on the species-specific duplications for *P. cultripes*. We detected 2,527 species-specific gene expansions, out of which 476 (18.8%) belonged to putative TEs. 1,039 of these expansions have a cluster size of 2 (i.e. they are formed by 2 in-paralogues), but more than 350 have a cluster size of 10 or higher (Supplementary Fig. S4), with some expansions reaching up to 1,040 (with the involvement of TEs).

**Figure 4:**
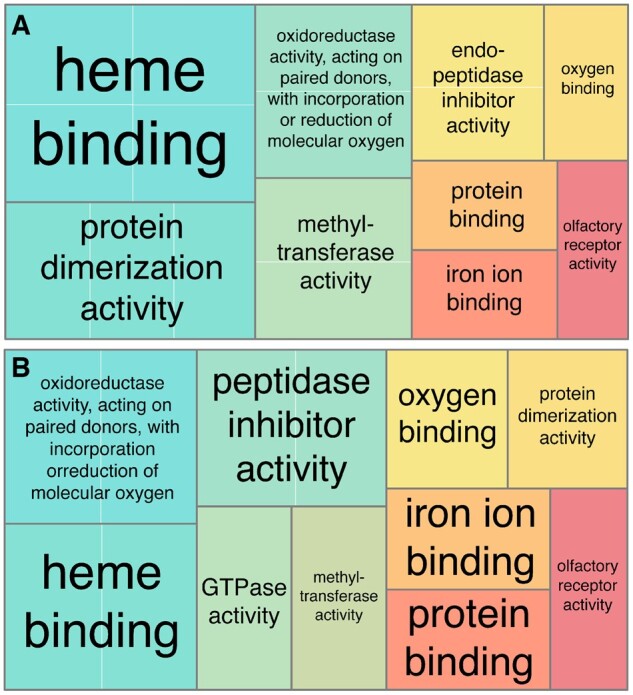
REVIGO tree maps summarizing enriched GO terms for molecular function in the *P. cultripes* genome. The size of the rectangles is relative to the significance (*P*-values) of each term in the enrichment analyses. Main terms are outlined in black and annotated, with ‘dispensable’, nested terms outlined in white. The tree maps show the differences in enriched terms composition (relative size and position) when (A) including versus (B) excluding expansions in the enrichment analysis.

Results from Gene Ontology (GO) term enrichment analysis of species-specific duplicated genes are consistent with previous studies,^85^ highlighting the importance of anuran skin respiration (associated to oxidoreductase, oxygen binding and haemo/iron binding) as well as with the metabolic activity (protein binding, protein dimerization, endopeptidase activity etc.). The exclusion of TEs from duplicated genes did not substantially change the overall GO term enrichment (Fig. 4).

### 3.3. Orphan gene composition in the western spadefoot toad genome

A total of 6,432 genes from the *P. cultripes* genome (30.7% of the TE-filtered gene set) did not have any homologs in the other taxa included in this study. Moreover, 5,409 (84%) of these genes had no hit during the functional annotation process performed with Blast2GO. While some orphan genes may indeed be newly formed genes with no known function, many may actually be fragments of real genes or represent annotation artefacts. In fact, the orphan genes are shorter, with an average of 2.44 exons per gene and 75% of them are mono-exonic. We also found that the average coding GC content of the orphan genes dataset is higher than for the entire set of annotations (50.74% vs. 45.77%), which may be indicative of higher gene fragmentation among them. From these statistics, it is hard to tell exactly how many of the orphan genes are real and how many are due to annotation artefacts, and we decided to explore their putative functions more deeply. Considering that our analyses include *L.ailaonicum* and *L. leishanense*, both part of a sister branch to our seed species (all part of the superfamily Pelobatoidea), these genes may be species or clade specific. To further explore this hypothesis, all orphans were searched by BLAST against the non-redundant RefSeq protein database. BLAST yielded 5,171 unique hits, which were filtered using as a criterion 33% query coverage and 1e-5 E-value. Significant hits with above 33% query coverage were considered as putative homologs (826 proteins) whereas the significant hits below the query coverage were considered as putative *de novo* genes (439 proteins).

**Figure 5:**
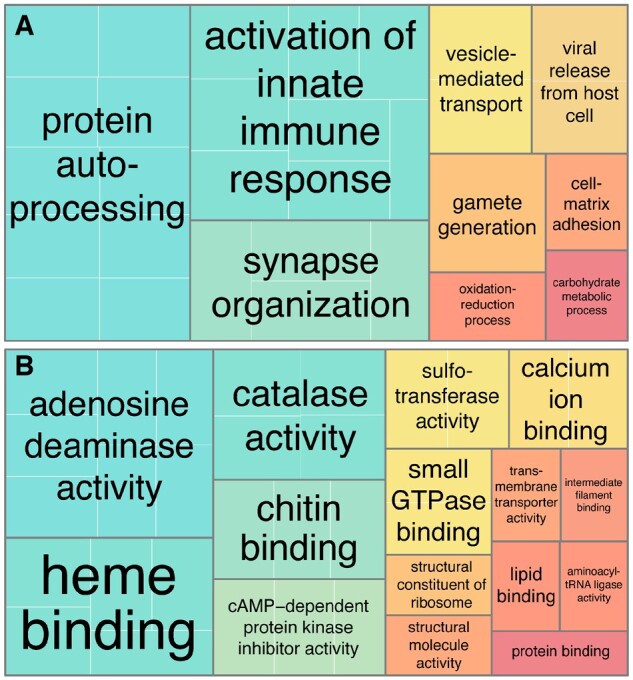
REVIGO tree maps summarizing GO terms in the orphan genes the *P. cultripes* genome. The tree maps show (A) biological process and (B) molecular function terms. The size of the rectangles is relative to the significance (*P*-values) of each term in the enrichment analyses. Main terms are outlined in black and annotated, with ‘dispensable’, nested terms outlined in white.

Domain-based functional annotation was only possible for 72 out of the original 6,432 genes, which yielded 172 GO annotations. This low presence of known domains is expected for *de novo* genes. The identified GO annotations were very diverse and general (Fig. 5). The appearance of GO terms assigned to immune response is to be expected; however, viral release from host cells and chitin binding seem to correspond to a retroviral element inserted in the genome (LTR) that partially escaped the masking. Some terms are also common in other anurans (i.e. Heme binding), whereas others are a bit more surprising such as gamete generation and synapse organization. Of these 72 proteins, 37 were classified according to our criteria as putative homologs, and 31 were classified as putative *de novo* genes.

## 4. Conclusions

The combination of Illumina, Nanopore and Hi-C sequencing technologies has resulted in a near-complete-chromosome-level genome assembly for the western spadefoot toad, *P.cultripes*. The assembly is extensively annotated using transcriptomic data and placed into an evolutionary context using phylogenomics. This high-quality genome assembly and the corresponding phylome represent important resources for vertebrate genomics, in which amphibians continue to be grossly under-represented.

## Supplementary data

Supplementary data are available at DNARES online.

## Supplementary Material

dsac013_Supplementary_Data
