# Supplementary material for: Chromosome-level assembly, annotation and phylome of Pelobates cultripes, the western spadefoot toad
Source: DNA Res. 2022 May 18;29(3):dsac013. doi: 10.1093/dnares/dsac013 (PMC9164646; doi:10.1093/dnares/dsac013)
Supplement: dsac013_Supplementary_Data [file dsac013_supplementary_data.zip › dsac013_Supplementary_Data/Supplementary figure legends_R1.docx]

**Supplementary figure and table legends:**

**Supplementary Figure 1.** Flow chart of the approach used to assemble the *Pelobates cultripes* genome.

**Supplementary Figure 2.** Flow chart of the approach used to annotate the *Pelobates cultripes* genome.

**Supplementary Figure 3.** Hi-C contact map of the 13 chromosome-length scaffolds, and an additional, unplaced 14^th^ scaffold, possibly a microchromosome. In this figure, the x and y axes give the mapping positions of the first and second read in the read pair respectively, grouped into bins. The intensity of the red colour of each square indicates the relative number of read pairs within that bin.

**Supplementary Figure 4.** Number of clusters *vs*. cluster size distribution of gene expansions.

**Supplementary Table 1.** Assembly statistics for different stages of the assembly process.

**Supplementary Table 2.** Species and NCBI taxon IDs for species included in the phylome analysis.

**Supplementary Table 3.** Lengths of superscaffolds of final assembly *Pelobates cultripes* assembly.
