# Supplementary figures and images for: Chromosome-level assembly, annotation and phylome of Pelobates cultripes, the western spadefoot toad
Source: DNA Res. 2022 May 18;29(3):dsac013. doi: 10.1093/dnares/dsac013 (PMC9164646; doi:10.1093/dnares/dsac013)

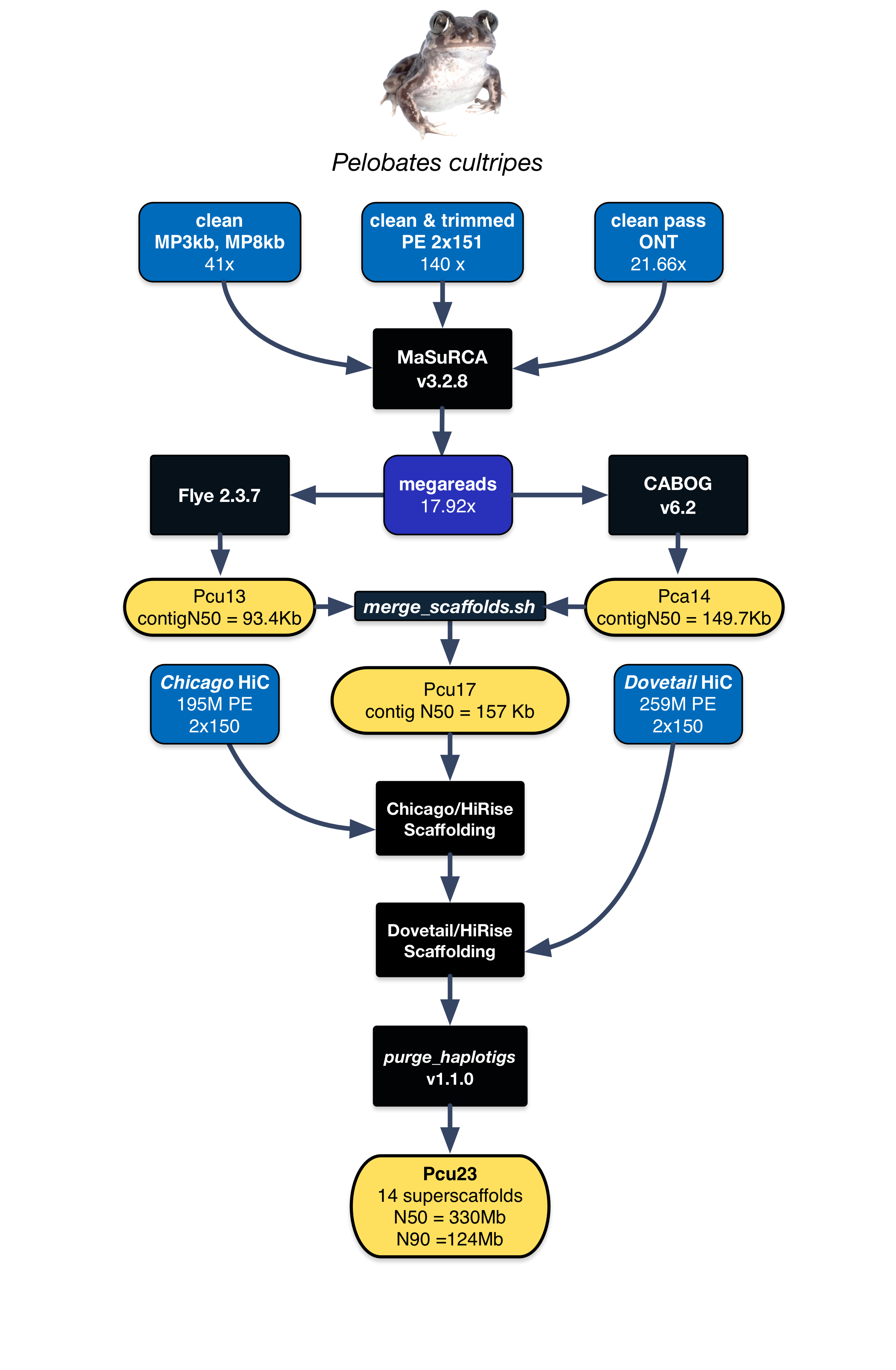

Supplement: dsac013_Supplementary_Data [file dsac013_supplementary_data.zip › dsac013_Supplementary_Data/SuppFig_1.jpg]

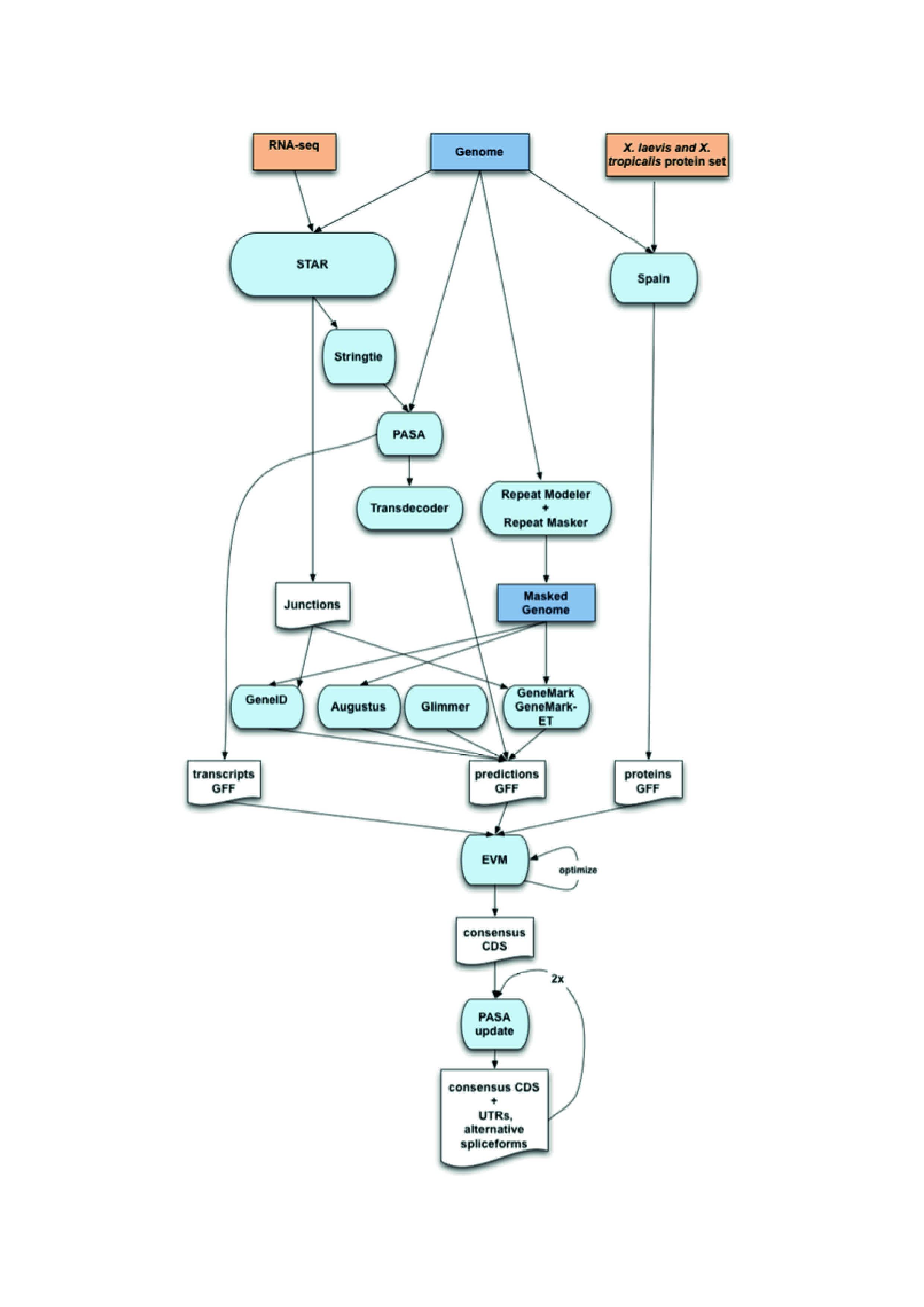

Supplement: dsac013_Supplementary_Data [file dsac013_supplementary_data.zip › dsac013_Supplementary_Data/SuppFig_2.jpg]

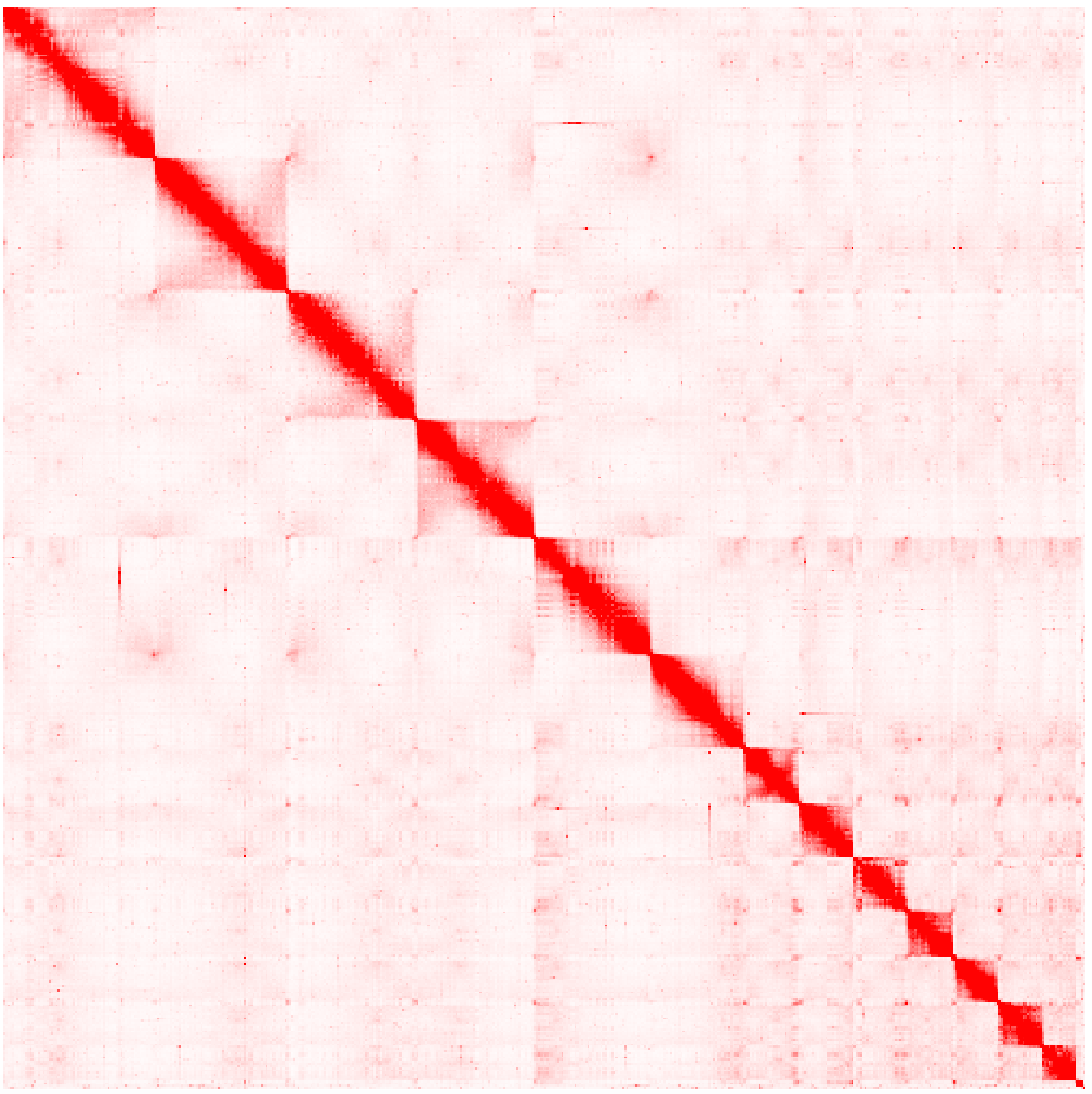

Supplement: dsac013_Supplementary_Data [file dsac013_supplementary_data.zip › dsac013_Supplementary_Data/SuppFig_3.png]

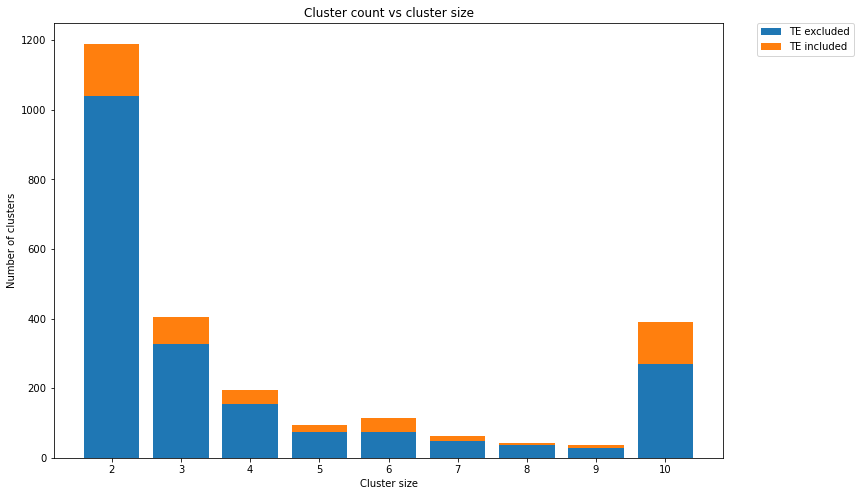

Supplement: dsac013_Supplementary_Data [file dsac013_supplementary_data.zip › dsac013_Supplementary_Data/SuppFig_4.png]
